# Empagliflozin Attenuates Non-Alcoholic Fatty Liver Disease (NAFLD) in High Fat Diet Fed ApoE^(-/-)^ Mice by Activating Autophagy and Reducing ER Stress and Apoptosis

**DOI:** 10.3390/ijms22020818

**Published:** 2021-01-15

**Authors:** Narjes Nasiri-Ansari, Chrysa Nikolopoulou, Katerina Papoutsi, Ioannis Kyrou, Christos S. Mantzoros, Georgios Kyriakopoulos, Antonios Chatzigeorgiou, Vassiliki Kalotychou, Manpal S. Randeva, Kamaljit Chatha, Konstantinos Kontzoglou, Gregory Kaltsas, Athanasios G. Papavassiliou, Harpal S. Randeva, Eva Kassi

**Affiliations:** 1 Department of Biological Chemistry, Medical School, National and Kapodistrian University of Athens, 11527 Athens, Greece; nnasiri@med.uoa.gr (N.N.-A.); chrysa_nikolopoulou@hotmail.com (C.N.); pap.katerina@hotmail.com (K.P.); geokyr11@hotmail.gr (G.K.); papavas@med.uoa.gr (A.G.P.); 2 Warwickshire Institute for the Study of Diabetes, Endocrinology and Metabolism (WISDEM), University Hospitals Coventry and Warwickshire NHS Trust, Coventry CV2 2DX, UK; kyrouj@gmail.com; 3 Aston Medical Research Institute, Aston Medical School, Aston University, Birmingham B4 7ET, UK; 4 Division of Biomedical Sciences, Warwick Medical School, University of Warwick, Coventry CV4 7AL, UK; 5 Division of Endocrinology, Diabetes and Metabolism, Beth Israel Deaconess Medical Center, Harvard Medical School, Boston, MA 02215, USA; cmantzor@bidmc.harvard.edu; 6 Section of Endocrinology, Boston VA Healthcare System, Harvard Medical School, Boston, MA 02215, USA; 7 Department of Pathology, Evangelismos Hospital, 10676 Athens, Greece; 8 Department of Physiology, Medical School, National and Kapodistrian University of Athens, 11527 Athens, Greece; achatzig@med.uoa.gr; 9 1st Department of Internal Medicine, Laiko Hospital, Medical School, National and Kapodistrian University of Athens, 11527 Athens, Greece; vkalotyc@med.uoa.gr; 10 Human Metabolism Research Unit, WISDEM Centre, NHS Trust, Coventry CV2 2DX, UK; manpalrandeva@hotmail.com; 11 Department of Biochemistry & Immunology, University Hospitals Coventry and Warwickshire NHS Trust, Coventry CV2 2DX, UK; kamaljit.chatha@uhcw.nhs.uk; 12 Laboratory of Experimental Surgery and Surgical Research N.S. Christeas, Athens University Medical School, National and Kapodistrian University of Athens, 11527 Athens, Greece; kckont@med.uoa.gr; 13 Endocrine Oncology Unit, 1st Department of Propaupedic Internal Medicine, Laiko Hospital, National and Kapodistrian University of Athens, 11527 Athens, Greece; gkaltsas@endo.gr; 14 Division of Translational and Experimental Medicine-Metabolic and Vascular Health, Warwick Medical School, University of Warwick, Coventry CV4 7AL, UK

**Keywords:** NAFLD, SGLT-2 inhibitors, autophagy, ER stress, apoptosis, inflammation

## Abstract

Aims/hypothesis: SGLT-2 inhibitors (SGLT-2i) have been studied as potential treatments against NAFLD, showing varying beneficial effects. The molecular mechanisms mediating these effects have not been fully clarified. Herein, we investigated the impact of empagliflozin on NAFLD, focusing particularly on ER stress, autophagy and apoptosis. Methods: Five-week old ApoE^(-/-)^ mice were switched from normal to a high-fat diet (HFD). After five weeks, mice were randomly allocated into a control group (HFD + vehicle) and Empa group (HFD + empagliflozin 10 mg/kg/day) for five weeks. At the end of treatment, histomorphometric analysis was performed in liver, mRNA levels of *Fasn*, *Screbp-1*, *Scd-1*, *Ppar-γ*, *Pck-1*, *Mcp-1*, *Tnf-α*, *Il-6*, *F4/80*, *Atf4*, *Elf2α*, *Chop*, *Grp78*, *Grp94*, *Χbp1*, *Ire1α*, *Atf6*, *mTor*, *Lc3b*, *Beclin-1*, *P62*, *Bcl-2* and *Bax* were measured by qRT-PCR, and protein levels of p-EIF2α, EIF2a, CHOP, LC3II, P62, BECLIN-1 and cleaved CASPASE-8 were assessed by immunoblotting. Results: Empagliflozin-treated mice exhibited reduced fasting glucose, total cholesterol and triglyceride serum levels, as well as decreased NAFLD activity score, decreased expression of lipogenic enzymes (*Fasn*, *Screbp-1c* and *Pck-1*) and inflammatory molecules (*Mcp-1* and *F4/80*), compared to the Control group. Empagliflozin significantly decreased the expression of ER stress molecules *Grp78*, *Ire1α*, *Xbp1*, *Elf2α*, *Atf4*, *Atf6*, *Chop*, *P62(Sqstm1)* and *Grp94*; whilst activating autophagy via increased AMPK phosphorylation, decreased *mTOR* and increased *LC3B* expression. Finally, empagliflozin increased the *Bcl2/Bax* ratio and inhibited CASPASE-8 cleavage, reducing liver cell apoptosis. Immunoblotting analysis confirmed the qPCR results. Conclusion: These novel findings indicate that empagliflozin treatment for five weeks attenuates NAFLD progression in ApoE^(-/-)^ mice by promoting autophagy, reducing ER stress and inhibiting hepatic apoptosis.

## 1. Introduction

Non-alcoholic fatty liver disease (NAFLD) is one of the most common causes of chronic liver disease worldwide [1,2]. NAFLD ranges from simple steatosis without inflammation to non-alcoholic steatohepatitis (NASH), which, in some cases, can lead to cirrhosis and even hepatocellular carcinoma [1,2]. Despite its high prevalence and morbidity, there is currently no approved therapy for NAFLD/NASH.

Higher prevalence of type 2 diabetes (DMT2) has been documented among NAFLD and NASH patients, while NAFLD also markedly increases the risk of developing DMT2 [3]. NAFLD and DMT2 share common pathophysiologic features, with insulin resistance playing a key pathogenic role [4]. As such, anti-diabetic drugs (e.g., metformin, thiazolidinediones and GLP-1 analogues) have been studied as potential treatments against NAFLD/NASH development and progression [5,6]. Sodium-glucose co-transporter-2 inhibitors (SGLT-2i) represent a new class of anti-diabetic drugs which act mainly through increasing urinary glucose excretion and, thus, improve glucose control independently of insulin secretion [7]. In addition to glucose reduction, SGLT-2i has also been shown to exert certain beneficial cardio-metabolic effects, including weight reduction and cardiovascular disease (CVD) protection [8,9,10].

Recently, a number of randomized and non-randomized clinical trials have reported beneficial effects of SGLT-2i on NAFLD, as assessed either by surrogate biomarkers/indices (e.g., alanine aminotransferase (ALT), aspartate aminotransferase (AST), gamma-glutamyl transferase (γ-GT), triglycerides, hepatic insulin sensitivity indices), or by intrahepatic fat content on CT, MRI and proton-magnetic resonance spectroscopy imaging [6,11]. Of note, a recent large, placebo-controlled randomized clinical trial, i.e., the EMPA-REG OUTCOME trial, further supported these results, showing that empagliflozin treatment for 24 weeks in patients with DMT2 significantly reduced glutamic-pyruvic transaminase (SGPT) levels (a surrogate biomarker of liver fat) independently of changes in haemoglobin A1c (HbA1c) and body weight [12]. Interestingly, this improvement was more pronounced in the empagliflozin-treated arm compared to glimepiride, suggesting direct empagliflozin-induced effects on NAFLD progression, irrespective of glycemic control [12]. The E-LIFT trial also showed that treatment with empagliflozin (10 mg daily) for 20 weeks significantly reduced liver enzymes and liver fat in in 50 patients with DMT2 and NAFLD [13]. Although such data indicate that SGLT-2i may constitute a promising treatment option against NAFLD/NASH, the molecular mechanisms mediating the beneficial effects of SGLT-2i on biochemical and/or histological NAFLD features remain incompletely explored.

Recently, endoplasmic reticulum (ER) stress and autophagy have emerged as important underlying mechanisms in NAFLD/NASH development and progression, both regulating hepatic cell apoptosis [14,15]. Indeed, it is now well-known that both hyperglycaemia and lipid accumulation can cause proteostasis and trigger ER stress in hepatocytes [16]. In turn, an adaptive signalling pathway, i.e., the unfolded protein response (UPR), is activated to restore it [16]. To that effect, UPR enhances ER protein folding and induces clearance of aggregate-prone proteins by promoting autophagy. Subsequently, autophagy stimulates the degradation of intracellular lipid droplets (lipophagy). However, under chronic ER stress the UPR turns from adaptive to terminal, leading to hepatocyte death by increasing inflammation, reducing autophagic processes and activating pro-apoptotic pathways [17].

Although a number of studies have investigated SGLT-2i effects on ER stress in renal tubular cells, cardiomyocytes and pancreatic β-cells [18,19,20] data on their role in ER stress related pathways in NAFLD are sparse [21], and even completely lacking regarding the potential impact of SGLT-2i in NAFLD-related autophagy processes. As such, the aim of the present study was to investigate the effect of empagliflozin in NAFLD progression, focusing specifically on ER stress, autophagy and apoptosis.

## 2. Results

### 2.1. Empagliflozin Administration for Five Weeks Improves Fasting Blood Glucose and Lipid Profiles

**Figure 1:**
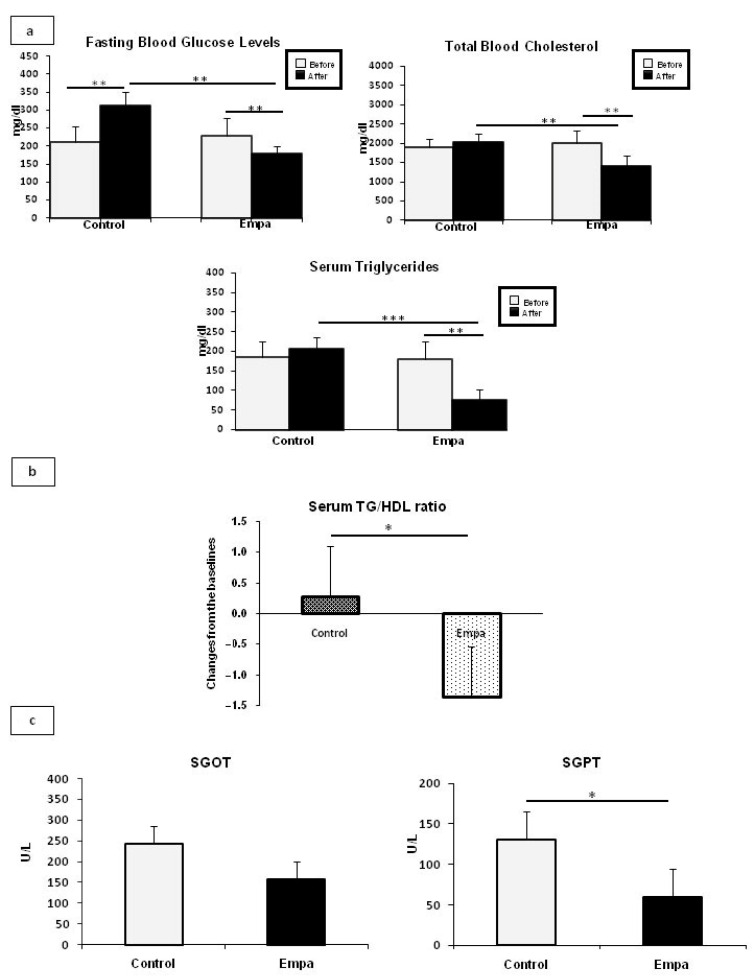
Serum fasting glucose, lipid, SGOT and SGPT concentrations in the Empa and control groups after five weeks of empagliflozin/vehicle oral administration. (**a**). A significant reduction in fasting blood glucose, total cholesterol, triglyceride levels was observed in the Empa group at the end of the treatment period compared to baseline. Fasting glucose was the only significantly increased parameter in the control group at the end of intervention as compared to baseline values. (**b**). Significant changes were detected from baseline in triglyceride/HDL ratio between two groups. (**c**). Serum SGOT and SGPT levels were reduced in Empa group as compared to Control group (*p* = 0.07 and *p* = 0.048, respectively) (*n* = 8 per group). Data are shown as the mean ± SD (***: *p* < 0.001; **: *p* < 0.01, *: *p* < 0.05).

No significant difference in daily food intake was observed between the two groups (*p* = 0.5). Empagliflozin administration had no significant effect on body weight as both HFD-fed ApoE^(-/-)^ mice groups significantly increased their body weight at the end of the five-week intervention compared to baseline (18.7% and 17.9% increase in body weight in the Empa and the control group, respectively). Empagliflozin treatment resulted in significantly reduced fasting glucose, total cholesterol, and triglyceride serum levels at the end of the five-week intervention compared to baseline (all *p*-values < 0.01). Additionally, fasting glucose, total cholesterol, and triglyceride serum levels were significantly lower in the Empa group compared to the control group at the end of the five-week intervention (*p* < 0.01, *p* < 0.01, and *p* < 0.001, respectively) (Figure 1a).

Recent data indicate that the triglyceride/HDL cholesterol ratio can be used as a new marker for prediction of endothelial dysfunction and as an indicator of increased risk of developing metabolic and cardiovascular complications in human [22]. To this end, we next measured the TG/HDL ratio in mice, and our result showed that at the end of Empagliflozin/placebo oral treatment, there was a significant difference from baseline in TG/HDL (*p* < 0.05) between groups (Figure 1b).

After completion of the five-week empagliflozin treatment, oxaloacetic transaminase (SGOT) levels were marginally decreased (*p* = 0.07), while a significant reduction in SGPT levels (*p* = 0.048) was observed in the Empa group as compared to the control group (Figure 1c).

### 2.2. Empagliflozin Administration for Five Weeks Improves Hepatic Lipid Accumulation

**Figure 2:**
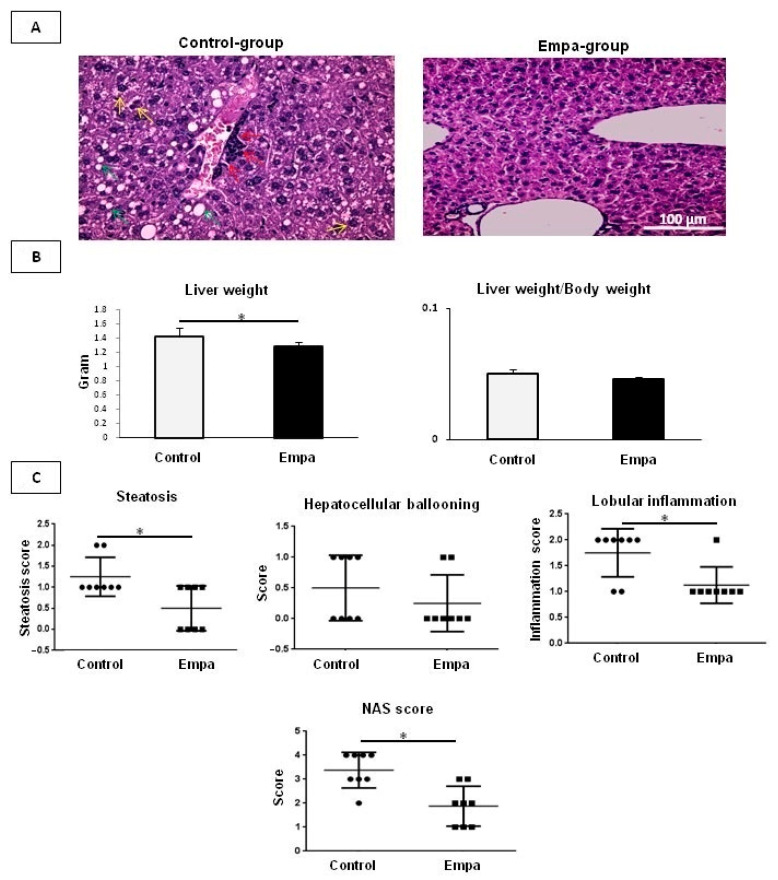
Histological assessment of NAFLD/NASH severity. (**A**) Representative images of H&E-stained slides of ApoE^(-/-)^ mice after five weeks of empagliflozin/vehicle oral administration. Lobular inflammation, ballooning cells and cytoplasmic lipid droplets are shown by red, yellow and green arrows, respectively. (**B**) The liver weight and the ratio of liver weight to body weight. (**C**) Histological evaluation of steatosis, hepatocellular ballooning, lobular inflammation and NAS score. Data are shown as the mean ± SD (*: *p* < 0.05).

ApoE mice in the control group had higher liver weights than the Empa group (*p* = 0.047); however, the liver weight to body weight ratio was not different (*p* = 0.2) between the two groups (Figure 2B).

The effect of empagliflozin/vehicle treatment on hepatic lipid accumulation and injury was evaluated in H&E staining. In the Empa group an overall beneficial effect was noted on steatohepatitis-related parameters, including decreased steatosis percentage, intrahepatic ballooning and lobular inflammation, thus leading to significantly improved liver histology (Figure 2A). As such, NAS was significantly lower in the Empa group compared to control (*p* = 0.04), attributed mainly to the significantly reduced lobular inflammation (*p* = 0.04) and steatosis (*p =* 0.04) (Figure 2C).

Of note, no liver fibrosis was detected at the end of intervention neither in the control nor Empa group (data not shown).

### 2.3. Empagliflozin Administration for Five Weeks Reduces the Expression of Lipogenic Enzymes and Inflammatory Markers

**Figure 3:**
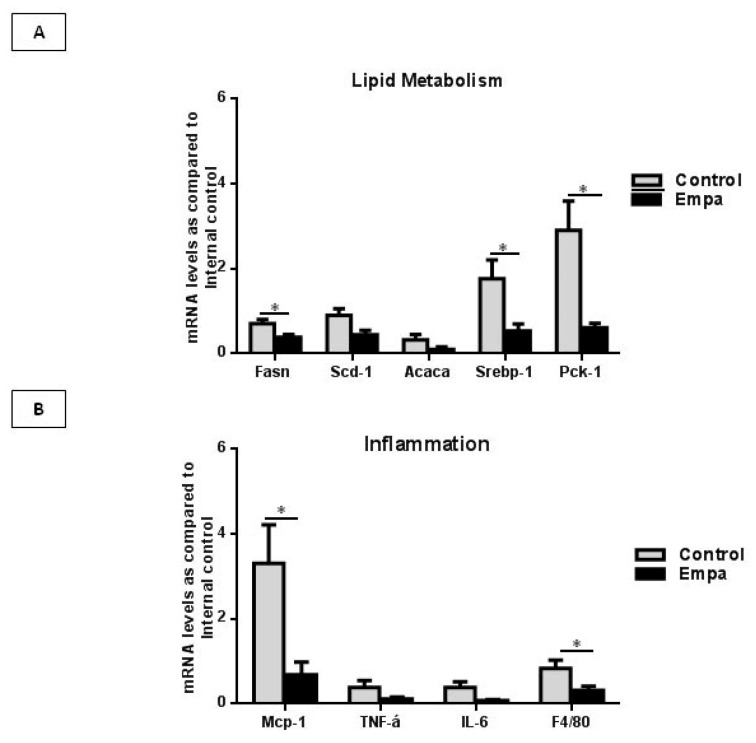
The hepatic expression of lipogenic enzymes and inflammatory markers in ApoE^(-/-)^ mice after five weeks of empagliflozin/vehicle oral administration. (**A**). The mRNA levels of *Fasn*, *Screbp-1* and *Pck-1* were significantly reduced in the Empa group as compared to the Control group. (**B**) Empagliflozin oral administration for five weeks resulted in significant reduction in *Mcp-1* and *F4/80* mRNA levels. Data are shown as the mean ± SEM (*: *p* < 0.05).

Peroxisome proliferator-activated receptor-gamma (*Ppar-γ*) is a known regulator of de novo lipogenesis (DNL) and its upregulation leads to the consequent lipid droplets deposition within hepatocytes. The *Srebp1c* plays a central role in controlling expression of genes involved in DNL such as *Acaca*, *Fasn* and *Scd-1*. *Pck1* is a regulator of re-esterification of free fatty acid into triacylglycerol [16,21]. As such, we evaluated whether empagliflozin had an impact on the hepatic lipogenesis pathway by measuring the expression of these lipogenic genes. The *Fasn*, *Srebp-1c* and *Pck-1* gene expression was significantly lower in Empa group as compared to the Control group (*p* = 0.03, *p* = 0.02, *p* = 0.02, respectively). A marginal reduction in the expression of *Acaca* and *Scd-1* was also observed in Empa group when compared to the Control group (*p* < 0.1) (Figure 3A). No difference was observed in *Ppar-γ* expression between the two groups (*p* = 0.2).

*Mcp-1* recruits and activates monocytes/macrophages to the site of tissue injury, and regulates the expression of pro-inflammatory cytokines, such as TNF-α and IL-6. F4/80 is a known marker of resident macrophages. Thus, we assessed the effect of empagliflozin on the expression of the aforementioned pro-inflammatory genes. Our results showed that the expression of *Mcp-1* and *F4/80* was significantly reduced by five weeks of empagliflozin administration (*p* = 0.02 in both cases). A borderline decrease in the expression of *TNF-α* and *IL-6* in the Empa group was observed, compared to the control group (*p* < 0.1) (Figure 3B).

Of note, the liver in all mice expressed *SGLT-1* mRNA levels without any significant difference between two groups, while *SGLT-2* mRNA was faintly detected in almost half of the animals in each groups.

### 2.4. Empagliflozin Administration for Five Weeks Reduces the Expression of ER Stress Markers

**Figure 4:**
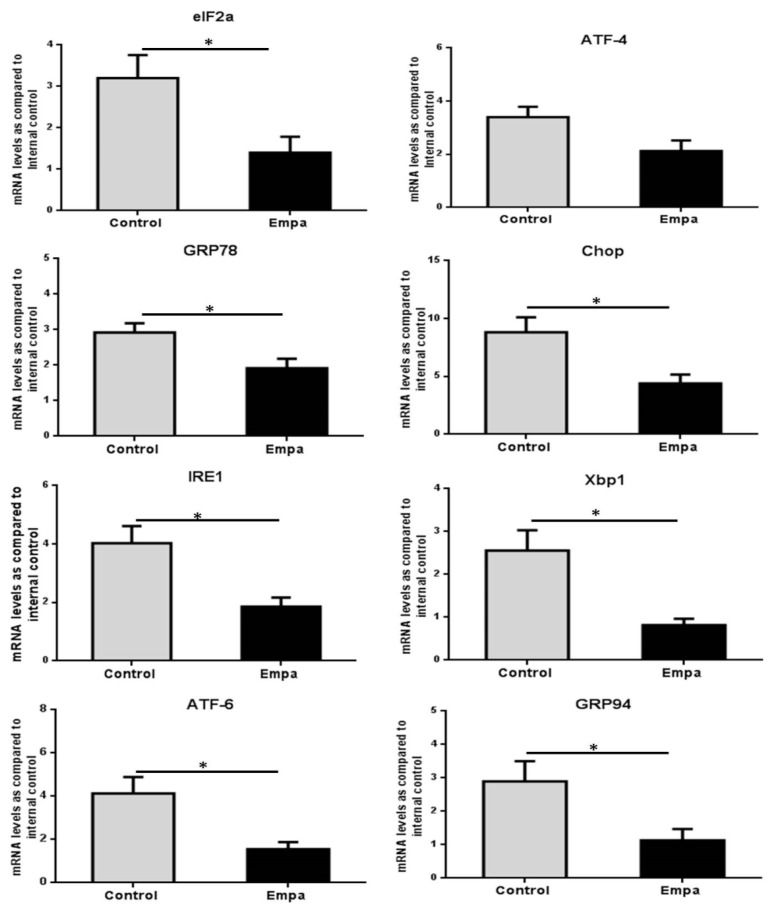
Changes of ER stress core genes mRNA levels after five weeks of empagliflozin/vehicle oral administration in the liver of ApoE^(-/-)^ mice. The mRNA levels of *elf2α*, *ATF-4*, *GRP78*, *CHOP*, *IRE1*, *XbP1*, *ATF-6* and *GRP94* were significantly reduced after five weeks of empagliflozin treatment. Data are shown as the mean ± SEM (*: *p* < 0.05).

The mRNA levels of factors involved in the regulation of the ER stress response (*GRP78*, *eIF2a*, *ATF4*, *IRE1*, *Xbp1*, *ATF-6*, *GRP94* and *CHOP*) were evaluated by qPCR, whereas protein levels of eIF2a, p-eIF2a and CHOP were evaluated by Western blot. Our results demonstrated that the five-week empagliflozin treatment resulted in significantly lower mRNA expression of *GRP94*, *eIF2α*, *GRP78*, *IRE1*, *Xbp1*, *CHOP* and *ATF6* compared to control (*p* = 0.03, *p* = 0.03, *p* = 0.03, *p* = 0.015, *p* = 0.02, *p* = 0.02 and *p* = 0.02, respectively) (Figure 4).

Moreover, marginally significant lower *ATF4* mRNA levels were also observed in the Empa group (*p* = 0.05) (Figure 4).

**Figure 5:**
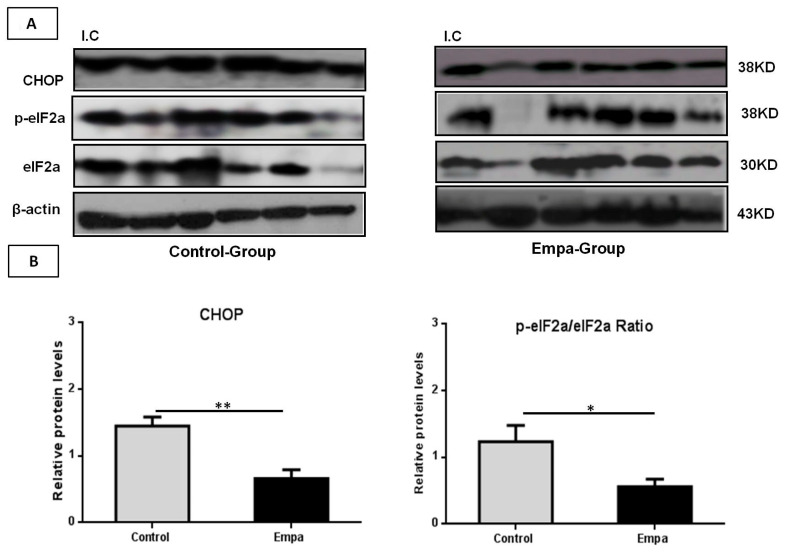
Empagliflozin administration for five weeks reduces protein levels of ER stress markers. (**A**). Western blot analysis of CHOP, total elf-2α and phospho elf-2α in liver tissues. (**B**) Quantitative analyses showed that empagliflozin reduces the protein levels of CHOP, p-elf-2α/elf-2α ratio. Data are expressed as the mean ± SEM (*: *p* < 0.05; **: *p* < 0.01; I.C: Internal control).

Western blot analysis revealed that CHOP protein levels, as well as the p-eIF2a/eIF2a protein levels ratio, were significantly lower in empagliflozin-treated mice compared to the control group (*p* = 0.004 and *p* = 0.04, respectively) (Figure 5A,B).

### 2.5. Empagliflozin Administration for Five Weeks Activates the Hepatic Autophagic Flux

**Figure 6:**
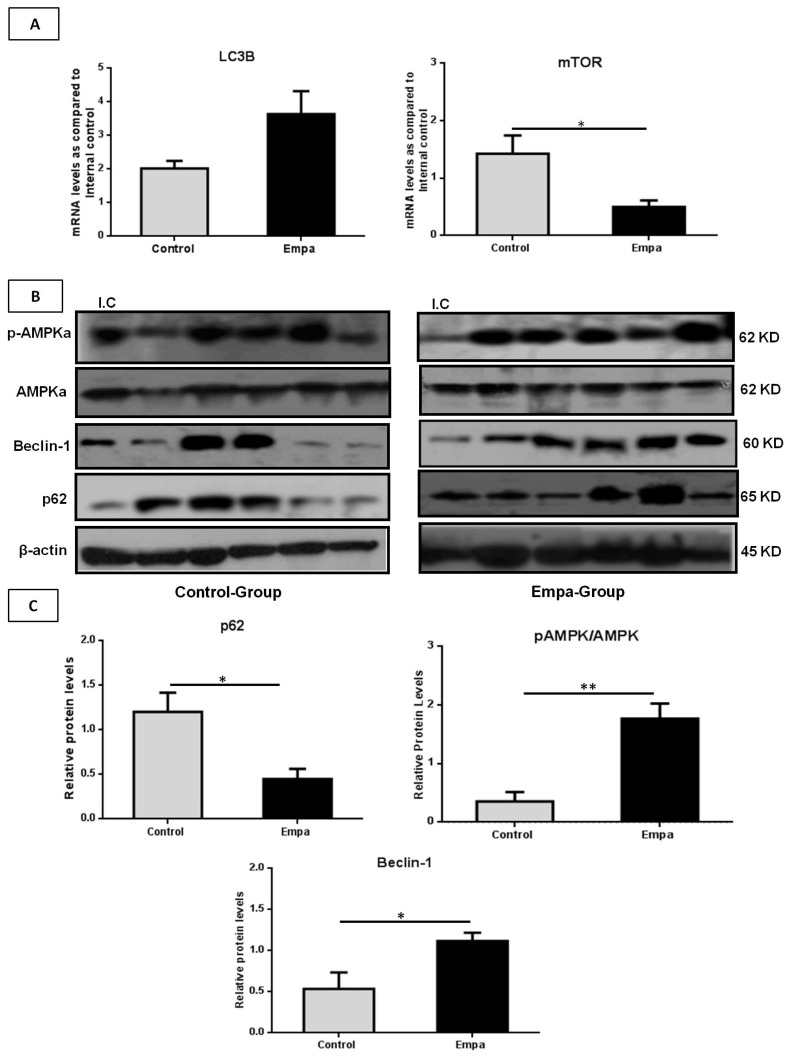
The mRNA and protein expression profiles of the autophagy-related genes. (**A**) Relative mRNA levels of *LC3B* was increased while the *mTOR* mRNA expression was reduced after five weeks of empagliflozin administration. (**B**) Western blot analysis of p62, Beclin-1, total AMPKa and phospho-AMPKa in liver tissues. (**C**) Quantitative analyses showed that empagliflozin reduces p62, while it increases the p-AMPKa/AMPKa ratio and Beclin-1 protein levels. Data are shown as the mean ± SEM (*: *p* < 0.05; **: *p* < 0.01; I.C: Internal control).

Both mRNA and protein levels of autophagy (macro-autophagy) markers were also assessed. Results of the qRT-PCR analysis demonstrated that, compared to the control group, the five-week empagliflozin treatment resulted in significantly lower *mTOR* mRNA levels (*p* = 0.03), while *LC3B* mRNA levels were higher in the Empa group almost reaching statistical significance (*p* = 0.06) (Figure 6A). No differences were observed in *Beclin-1*, *p62*, *AMPKa1* and *AMPKa2* mRNA levels between the two groups (*p* = 0.2, *p* = 0.5, *p* = 0.12 and *p* = 0.17, respectively) at the end of the five-week treatment period (data not shown).

However, compared to the control group, p62 protein levels were significantly lower in the Empa group (*p* = 0.04) at the end of this five-week intervention, while Beclin-1 and the ratio of phospho-AMPK/AMPK protein levels were significantly higher (*p* = 0.046 and *p* = 0.005, respectively) (Figure 6B,C).

### 2.6. Empagliflozin Administration for Five Weeks Attenuates Hepatic Apoptosis

**Figure 7:**
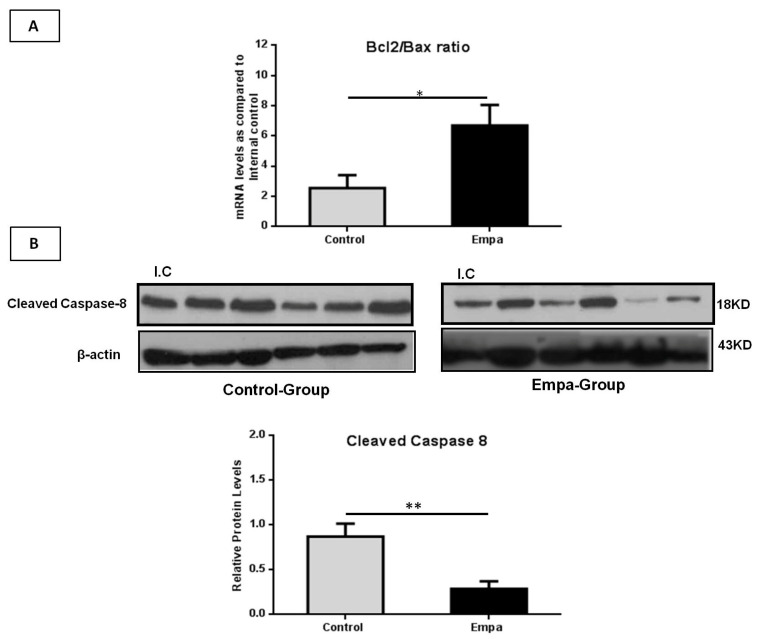
The mRNA and protein levels of apoptosis related molecules. (**A**) Empagliflozin treatment for five weeks significantly increased the mRNA *Bcl2/Bax* ratio. (**B**) Western blot analysis of cleaved caspase-8 with quantitative analyses showing that empagliflozin reduces the protein levels of cleaved caspase-8. Data are shown as the mean ± SEM (*: *p* < 0.05; **: *p* < 0.01; I.C: Internal control).

The *Bcl-2/Bax* ratio is a known indicator of cell susceptibility to apoptosis, thus, we quantified both *Bcl-2* and *Bax* mRNA levels. Our results show that the *Bcl-2/Bax* ratio was significantly higher in the Empa group (*p* = 0.02) compared to the control group at the end of the five-week treatment (Figure 7A). In addition, at this time point, the protein expression of cleaved caspase-8 was found to be significantly lower in the Empa group compared to the control group (*p* = 0.006) (Figure 7B).

## 3. Discussion

The therapeutic potential of SGLT-2i on NAFLD has been previously reported in animal and in human studies [21,23,24,25,26,27,28]. To shed light on the underlying molecular mechanisms, we present here novel data showing that empagliflozin treatment for five weeks in HFD-fed ApoE^(-/-)^ mice not only decreases fasting glucose, total cholesterol and triglycerides serum levels, but also improves the underlying NAFLD histological features by activating autophagy, alleviating ER stress, and attenuating apoptosis. In the context of this study, HFD-fed ApoE^(-/-)^ mice were used as a NAFLD model since a high-fat, cholesterol-rich diet has been shown to accelerate development of NASH with fibrosis in this animal model [29,30].

Of note, in contrast to human studies [31], in the present study empagliflozin did not reduce the body weight in the treated mice compared to control. Indeed, both ApoE^(-/-)^ mice groups consumed similar amounts of food and gained weight without significant differences between them during the five-week treatment period of our study. This is in accord with previous studies from our group which also showed that canagliflozin and empagliflozin administration for 5 and 10 weeks, respectively, did not promote weight loss in ApoE deficient mice under HFD conditions [9,10]. Interestingly, conflicting results exist in the relevant literature with respect to SGLT-2i effects on body weight in animal models. As such, previous studies have showed that empagliflozin did not affect significantly the body weight of Zucker diabetic fatty (ZDF) rats (a type 2 diabetes animal model) at doses of 10 and 30 mg/kg/day, whereas it can decrease the body weight in ApoE^(-/-)^ mice at a dose of 1–3 mg/kg/day for eight weeks [32,33]. Recently, Petito da Silva et al. reported that empagliflozin administration at a dose of 10 mg/kg/day for five weeks results in decreased body weight in HFD-fed male C57Bl/6 mice [21]. The divergent results in existing animal studies regarding the effects of SGLT-2i on body weight could be attributed, at least in part, to differences in the used animal models, doses and durations of treatment, and underlying diets.

In addition to lowering fasting blood glucose levels, our data further show that in HFD-fed ApoE^(-/-)^ mice empagliflozin can also reduce total cholesterol and triglyceride serum levels over a five-week treatment period. This is in agreement with previous studies from our group and others conducted in various animal models (e.g., ob/ob or wild type mice) and with varying treatment regimens, which showed similar empagliflozin-induced effects on fasting lipid profiles [9,34]. Moreover, in line with our findings, recent studies demonstrated that administration of empagliflozin for 5 and 10 weeks at a dose of 10 mg/jg and 1.5 mg/jg/day, results in significantly reduced serum SGPT levels of HFD-fed male C57BL/6 and ob/ob^(-/-)^ mice, respectively [21,34].

It must be highlighted that, histological findings in the context of the present study showed a marked reduction of liver steatosis, lobular inflammation, ballooning and hepatocellular degeneration in the empagliflozin-treated group, resulting in significantly improved NAS compared to the control group. Similar improvements in hepatic steatosis and steatohepatitis have also been reported in other animal studies with administration of different SGLT-2i, including luseogliflozin, empagliflozin, remogliflozin, ipragliflozin and NGI001 [21,23,24,25,26,27]. Based on these studies, key mechanisms which appear to mediate these effects involve mainly the regulation of insulin resistance/glucose tolerance and changes in the expression of enzymes implicated in beta-oxidation and hepatic de novo lipogenesis [21,23,24,25,26,27]. In line with most of these studies, we found a reduction in the expression of six key-enzymes of lipogenesis which was more pronounced for Screbp-1c, Fasn and pck-1. Of note, Petito da Silva et al. recently reported reduced liver mRNA expression of *Srebp1c*, *Ppar-γ*, *Acc1*, *Scd-1* and *Fasn* in male C57Bl/6 mice treated with empagliflozin for five weeks [21].

The reduction of lipogenesis enzymes following empagliflozin administration could explain—at least in part—the decreased steatosis and fatty droplets area observed within hepatocytes. Accordingly, we found a decreased expression of inflammatory markers, such as *Mcp-1* and *F4/80*, and to a lesser extent *TNF-α* and *IL-6*, with empagliflozin treatment. The decrease in the expression of the aforementioned inflammatory indices has been shown to reduce macrophage infiltration and lobar inflammation in liver leading to alleviation of hepatic steatosis and inflammation [21,23,24,26].

Notably, the present study further expanded on the mechanisms underlying such empagliflozin-induced beneficial effects on NAFLD/NASH, focusing on the gene and protein hepatic expression of factors involved in ER stress, autophagy and apoptosis which are crucial processes implicated in NAFLD development and progression [35,36,37,38,39]. Indeed, it is now well-established that under normal (unstressed) conditions, IRE1, PERK and ATF6 are inactivated upon binding to GRP78/BiP, whereas under stress conditions GRP-78/BiP dissociates from the ER stress sensors, thus activating the three arms of the UPR [37]. Here, we found reduced expression of GRP78/BiP in the liver of the empagliflozin-treated ApoE^(-/-)^ mice compared to control. Overall, existing data regarding GRP78/BiP expression in NAFLD are conflicting [35,36]. Of note, Jo et al. [40] showed that tunicamycin, which can induce liver steatosis, increases GRP78/BiP expression. Moreover, dietary-induced obese mice have been shown to express higher hepatic GRP78/BiP levels compared to lean counterparts [41]. It might be expected here that, since GRP78 is a chaperone responsive to glucose, empagliflozin should decrease its expression through reducing glucose levels. However, using liver tissue from obese mice and cultured rat liver cells, Ozcan et al. found that hyperglycemia did not induce GRP78 expression, suggesting that its regulation is not likely related, at least exclusively, to glycemia [41].

Moreover, we showed here that the three branches of the UPR adaptive pathway, i.e., *PERK*, *IRE1a* and *ATF6*, were attenuated in the liver tissues of the empagliflozin-treated group compared to control. Specifically, we noted suppression of the PERK-elf2a-ATF4 arm in the liver, as indicated by the decreased phosphorylation of the elf protein and decreased mRNA expression of the transcription factor ATF4. Notably, this pathway is known to regulate lipogenesis and steatosis with ATF4-deficient mice exhibiting decreased synthesis of fatty acids and lower triglyceride serum levels [14].

Furthermore, our results revealed suppression of the IRE1a pathway in the empagliflozin-treated group, as indicated by the decreased mRNA expression of both *IRE1a*, and *Xbp1* which is the transcription factor activated by IRE1a. The IRE1a pathway is also an important regulator of hepatic lipogenesis. Indeed, Xbp1 ablation has been reported to ameliorate liver steatosis and injury, as well as hyperlipidemia in ApoE^(-/-)^ mice [42]. Lee et al. showed that selective deletion of Xbp1 in the liver led to decreased hepatic production of lipids through down regulation of critical lipogenic genes, such as Scd-1, diacyl glycerol acetyltransferase 2 (Dgat2) and acetyl coA carboxylase 2 (Acc2), and subsequently to marked hypocholesterolemia and hypotriglyceridemia [43]. Recently, it has been shown that activation of UPR-IRE-Xbp-1 can trigger activation of the Srebp-1c signalling pathway, leading to increased liver steatosis [44]. Therefore, the decreased activation of both PERK-elf2a-ATF4 and IRE1a-Xbp1 pathways could substantially contribute to the lower total cholesterol and triglyceride serum levels noted with empagliflozin treatment. Similarly, our findings further show that ATF6 expression is also reduced in the liver of the empagliflozin-treated ApoE^(-/-)^ mice compared to control. A recent study by Chen et al. also showed that down-regulation of the ATF6 signalling pathway alleviates the progression of NAFLD by inhibiting ER stress-induced inflammation and apoptosis of liver cells [45].

Interestingly, ATF4, Xbp1 and ATF6 pathways are known to activate UPR target genes implicated in autophagy and apoptosis through regulating CHOP [14]. In our study, the applied five-week empagliflozin treatment resulted in lower mRNA and protein CHOP levels compared to controls. Currently, there are only a few studies exhibiting beneficial effects of SGLT-2i (dapagliflozin and ipragliflozin) on ER stress markers, mostly relating to renal tubular cell inflammation injury in diabetic mice [19,20]. Empagliflozin has also been found to induce a protective effect against diabetic cardiomyopathy by inhibiting IRE1a-Xbp1 and ATF4-CHOP pathways [46], whilst it can also improve β-cell mass in streptozotocin-treated mice through down-regulation of Xbp1, BiP and ATF4 [18]. To the best of our knowledge, there is only one previous study investigating the effects of empagliflozin on ER stress in a diet-induced NAFLD mice model [21]. Indeed, in accord with our findings, Petito da Silva et al. have shown that empagliflozin can mitigate NAFLD development by reducing the expression of genes involved in the elf2α-ATF4-CHOP-GADD45 pathway of UPR activation [21].

As ER stress is a well-established regulator of autophagy via mainly ATF4 and Xbp-1 [17] it is also important to explore autophagic processes in the context of NAFLD. Autophagy breaks down, among others, the intracellular lipids in hepatocytes through a degradation pathway known as lipophagy [20]. Moreover, impaired autophagic flux in the liver is closely related to the development of hepatic steatosis [47]. Overall, the process of autophagosome formation involves three major steps, namely initiation which is mediated by AMPK activation; nucleation with the Beclin-1/class III PI3K complex; and elongation of the isolation membrane with the help of LC3 lipidation [47]. As such, we investigated the effect of empagliflozin on these autophagic processes. Although we noted no significant increase in the mRNA expression of both isoforms of the catalytic subunit of AMPK (AMPKa1 and AMPKa2), our findings demonstrate increased phosphorylation at the N-terminus of the a subunit (Thr172) of AMPK in the liver of empagliflozin-treated ApoE^(-/-)^ mice. The latter is required for full activation of AMPK. In line with our findings, a recent study by Xu et al. reported that administration of empagliflozin for 16 weeks in HFD-induced obese mice increased the phosphorylation of AMPK [48].

It is known that activated AMPK promotes autophagy through various mechanisms, including negative regulation of mTORC1 [49]. Interestingly, we found decreased *mTOR* mRNA expression with empagliflozin treatment, and increased Beclin-1 protein levels which promote the step of nucleation. Furthermore, out data indicate that empagliflozin upregulates the expression of LC3B (both at mRNA and protein level) which is also necessary for the autophagosome formation. In addition, we assessed liver P62/SQSTM1 protein levels which is a selective substrate of autophagy, usually used as an index of impaired autophagic flux [50]. P62/SQSTM1 protein levels were lower in the empagliflozin-treated mice of our study, indicating activation of autophagic flux by this SGLT-2i. It could be argued that the effects of empagliflozin can be attributed primarily to the decrease in glucose levels which could activate the energy-sensor AMPK, causing, in turn, the mTOR-mediated inhibition of autophagy. However, the noted glucose level decrease was not to a degree that can trigger this AMPK-induced mechanism, which is mainly activated under starvation conditions [51].

Taken together, these findings suggest that empagliflozin can activate all these steps of the autophagy process in the liver of HFD-fed ApoE^(-/-)^ mice.

Of note, these findings appear to partly contradict the existing knowledge that decreased ER stress leads to a diminished autophagy [52]. However, it should be noted that SGLT-2i has been found to primarily induce autophagy by a mechanism which has not been fully clarified. Indeed, in the myocardium and immune cells SGLT-2i appear to promote a state that resembles nutrient and oxygen deprivation [53,54]. This state can trigger -among other pathways- AMPK activation which is a main regulator of autophagy [55]. Similarly, compared to control, higher AMPK phosphorylation in the liver of empagliflozin-treated ApoE^(-/-)^ mice was also noted in our study. Additional studies are still required to fully clarify the SGLT-2i-induced effects on autophagy in the liver and other tissues.

Considering that both ER stress and autophagy can eventually regulate apoptosis, here we also investigated the effect of empagliflozin in hepatic apoptosis processes, showing that empagliflozin treatment leads to higher *Bcl-2/Bax* ratio and inactivated caspase-8 in the liver of HFD-fed ApoE^(-/-)^ mice. The former is a well-known apoptosis switch, with a progressive reduction of the *Bcl-2/Bax* ratio having been reported during the progression of NAFLD to NASH which correlates to the apoptosis percentage of hepatocytes [56]. Of interest, CHOP, which according to our results was lower with empagliflozin treatment, has been shown to inhibit Bcl-2 and up-regulate Bax, thus triggering the intrinsic apoptotic pathway [57].

Similarly, the lower hepatic caspase-8 cleavage levels we noted in empagliflozin-treated mice compared to control have also important implications for apoptotic pathways, since activated caspase-8 is required for extrinsic apoptosis and plays a crucial role in Free fatty acid (FFA)-mediated apoptosis in hepatocytes [58]. Interestingly, hepatocyte-specific caspase-8 knockout mice fed a methionine choline-deficient diet (a frequently-used nutritional NASH model) have been shown to exhibit decreased apoptosis and inflammatory processes [59].

Autophagy could also inhibit apoptosis via elimination ofp62/SQSTM1, which was found to be lower in the empagliflozin-treated mice of the present study. Of note, SQSTM1/p62 is not only an autophagy-specific substrate, but, when is abnormally accumulated, it can also stimulate the production of reactive oxygen species (ROS) and activate the DNA damage response [60]. As such, our results suggest that activation of autophagy by empagliflozin could directly inhibit hepatic apoptosis by both decreasing caspase-8 activation and eliminating SQSTM1/p62. However, the diminished ER stress (in particular the attenuation of PERK-elfa-ATF-4-CHOP pathway) could also mediate the decreased activation of caspase-8 [61]. The relative contribution of each of these pathways remains unclear and requires further study.

In addition, the reduced ER stress via CHOP could also directly inhibit apoptosis through increasing the Bcl-2/Bax ratio. Thus, it can be hypothesized that a potent autophagic flux due to empagliflozin treatment may attenuate the adaptive HFD-induced ER stress of hepatocytes, in a negative feedback loop. As such, both increased autophagy and reduced ER stress could eliminate hepatic apoptosis and alleviate NAFLD progression. Indeed, it has been demonstrated that autophagy can reduce ER stress, and so it can act protectively by inhibiting apoptosis through caspase inactivation [62]. Of further interest, a study by Zhou et al. in HFD-fed mice showed that inhibition of Scd-1 (a key enzyme in lipid metabolism) enhanced AMPK activity and autophagy (lipophagy), leading to decreased hepatic steatosis [63]. Emphasizing the role of AMPK in hepatic lipogenesis, another recent study documented that AMPK-dependent phosphorylation of insulin-induced gene (Insig) can prevent the activation of Srebp-1c and, consequently, its lipogenic enzymes which regulate triglyceride and fatty acid synthesis, including ATP citrate lyase (ACLY), glycerol-3-phosphate acyltransferase (GPAT), ACC1 and DGAT2 [64]. It becomes evident that, whether empagliflozin can act directly on AMPK which can initiate autophagy and regulate de novo lipogenesis, or on other molecules upstream of AMPK (e.g., Scd-1) remains to be further explored.

Finally, in support of our hypothesis that empagliflozin-triggered increased autophagy can decrease ER stress-induced NAFLD progression, Carloni et al. have demonstrated that rapamycin-induced autophagy inhibits ischemia-induced ER stress, thus protecting against brain injury [65,66]. In this context, administration of 3-methyadenine (an autophagy inhibitor) reversed this beneficial effect and accelerated the ER stress-induced neuronal death [65,66].

Whether the effects of empagliflozin on autophagy, ER stress and apoptosis are mediated directly through SGLT-2 inhibition in the liver tissue is a matter of great scientific interest. According to our data, SGLT-2 was low-expressed in half of the liver samples, without differences in the expression between the control group and Empa group. Interestingly, Obara et al. demonstrated the protein expression of SGLT-2 in human hepatocytes and hepatoma cell lines [67]. More interestingly, SGLT-1 was expressed in the total of the liver tissues (both control and intervention group) suggesting its possible role in the attenuation of NAFLD in our model. Of note, empagliflozin can also bind to SGLT-1, albeit with a lower affinity than SGLT-2; strengthening the suggestion that empagliflozin could act, among others, through SGLT-1, dual SGLT-1/SGLT-2 inhibitor phlorizin recently found to ameliorate NAFLD in Type 2 diabetic mice [68]. Nevertheless, the existence of other, unknown yet, protein(s) which could be a direct target of empagliflozin in hepatocytes cannot be excluded.

Expanding on our previous data [9], we also demonstrated that empagliflozin results in the reduction of pro-atherogenesis markers in aorta, including inflammatory markers (*Il-6*, *Tnf-α*), adhesion molecules (*Vcam-1*) and gelatinolytic activity (*Timp-1/Mmp-2* ratio), although no significant effect on atheroma plaque formation is present at the end of this five-week treatment period (Supplementary Data S1). This is in line with results which we have previously shown, where empagliflozin administered for 10 weeks in ApoE^(-/-)^ mice attenuates the progression of atherosclerotic plaque [9]. Notably, the strong bidirectional relationship between NAFLD and CVD is well established. Indeed, Kim et al. recently showed that NAFLD has prognostic value for identifying individuals who are at higher risk for CVD [69].

A limitation of our study is the lack of body composition assessments of the studied mice. However, the noted changes in total body weight during the five-week study intervention did not differ significantly between the empagliflozin-treated and control groups. Another study limitation is the lack of insulin sensitivity assessments before and after the empagliflozin treatment. Based on the study by Petito da Silva et al., empagliflozin has been shown to increase insulin sensitivity when given for five weeks in diet-induced NAFLD in C57Bl/6 mice [21].

## 4. Materials and Methods

### 4.1. Animals

Male C57BL/6J apolipoprotein E (ApoE) knockout mice (ApoE^(-/-)^) were originally purchased from “The Jackson Laboratory” and were bred in the animal facility of the National and Kapodistrian University of Athens. All animals were kept at a specific pathogen free (SPF) controlled environment (22–26 °C temperature, 40–60% humidity and 12 h light/dark cycle), with free access to water and regular chow diet until the age of five weeks. All animal experiments were approved by the local Animal Care and Use Committee (366495-09/07/2019).

### 4.2. Experimental Protocol

Male ApoE^(-/-)^ mice (*n* = 16) were used for the study experiments. At five weeks of age, all mice were switched to a high-fat diet (HFD: 20–23% by weight; 40–45% kcal from fat), containing cholesterol (0.2% total). After five weeks on this HFD, the ApoE^(-/-)^ mice were randomly divided into the following two groups: (1) Empagliflozin group (10 mg/kg/day empagliflozin, *n* = 8), and (2) control group (same volume of 0.5% hydroxyl ethylcellulose per day, *n* = 8). Empagliflozin or vehicle was administered orally by gavage daily for a period of five weeks. Empagliflozin was purchased from MCE (Cat.No.HY-15409) and dissolved in 0.5% hydroxyl ethylcellulose. During the five-week study treatment, food intake and body weight were measured once weekly. Blood glucose levels were also measured after 8–10 h fasting via tail puncture at baseline (before empagliflozin/vehicle oral administration), and at the study endpoint. At the end of the five-week study treatment, all mice were sacrificed under isoflurane anaesthesia by transection of the diaphragm and the liver and aorta was rapidly excised.

### 4.3. Serum Analysis of Biochemical Parameters

Venipuncture was performed once at baseline from the facial vein and once by heart puncturing after culling. Serum glucose, total-cholesterol and triglycerides were determined using appropriate enzymatic kits (Biosis TM, Athens, Greece) in a dedicated autoanalyzer.

### 4.4. RNA Extraction and Quantitative Real-Time PCR

Total RNA was extracted from fresh frozen liver tissues using NucleoSpin RNA Plus kit (Macherey-Nagel, Düren, Germany). The concentration and quality of extracted mRNA were evaluated by a NanoDrop™ instrument (Thermo Scientific, Waltham, MA, USA). Extracted RNA (1000 ng) was reverse transcribed into cDNA using LunaScript^®^ RTsynthesis kit ((New England Biolabs, Ipswich, MA, USA). Real-time PCR analysis was performed as described previously [10]. Expression of key regulatory molecules involved in lipogenesis such as fatty acid synthase (*Fasn*), sterol regulatory element-binding protein 1 (*Screbp-1*), acetyl CoA carboxylase (*Acaca*), stearoyl-CoA desaturase 1 (*Scd-1*), Peroxisome proliferator-activated receptor gamma (*Ppar-γ*) and phosphoenolpyruvate carboxykinase 1 (*Pck-1*), inflammatory markers such as monocyte chemoattractant protein-1(*Mcp-1*), tumour necrosis factor alpha (*TNF-α*), interleukin 6 (*IL-6*) and EGF-like module-containing mucin-like hormone receptor-like 1(*F4/80*), molecules involved in UPR regulation including activating transcription factor 4 (*ATF4*), binding immunoglobulin protein (*GRP78*), IRE1, eukaryotic initiation factor 2 alpha (*eIF2a*), X-box binding protein 1 (*Xbp1*), glucose-regulated protein 94 (*GRP94*), activating transcription factor 6 (*ATF6*) and C/EBP homologous protein *(CHOP*), as well as molecules involved in the regulation of autophagy, such as microtubule-associated protein 1 light chain 3B (*LC3B*), phosphoenolpyruvate carboxykinase 1 (*p62/SQSTM1*), mechanistic target of rapamycin kinase (*mTOR*), *Beclin-1*, AMP-activated protein kinasesubunit alpha-1 (*AMPKa1*), AMP-activated protein kinasesubunit alpha-2 (*AMPKa2*) acetyl CoA carboxylase (*Acaca*), apoptosis markers such as B-cell lymphoma 2 (*Bcl-2*) and BCL2-associated X protein (*Bax*), and the hepatic mRNA levels of *SGLT-1* and *SGLT-2* were measured using Luna^®^ Universal qPCR Master Mix (New England Biolabs, Ipswich, MA, USA) on a CFX96 (Bio-RAD, Hercules, CA, USA). A melting curve analysis was performed to confirm the specificity of quantitative polymerase chain reaction (qPCR) products. Fold-changes were calculated using the 2−ΔΔCt method and all values were normalized against 18 s mRNA expression. Differentially expressed genes were identified through fold change filtering where a minimum of two-fold change was considered significant. All reactions were performed in triplicates and repeated at least three times. The sequences of primers used for RT-PCR analysis in this study are listed in Supplementary Table S1.

### 4.5. Liver Histological Analysis

Mouse liver tissues were fixed in 10% neutral buffered formalin and embedded in paraffin blocks. The 4 μm-thick sections were stained with hematoxylin–eosin (H&E) (Sigma-Aldrich, St. Louis, MO, USA) and used for histopathological analysis while liver fibrosis was evaluated by Masson’s trichrome staining. For NAFLD/NASH diagnosis, percentage of steatosis, quantification of lobular inflammation and presence of hepatocellular degeneration were measured according to the NAFLD activity score (NAS). NAS scoring was performed in a blinded manner by two independent pathologists. On average three (2–4) tissue sections from each animal were used for histopathological evaluation.

### 4.6. SDS-PAGE and Western-Blot Analysis

Western blot analysis was performed as previously described. Briefly, whole protein was extracted from 60 mg of liver tissue using 2× lysis buffer (Cell Signalling Technology, MA, USA) supplemented with PMSF. Samples containing 30 μg of protein were resolved by electrophoresis gels and transferred to a PVDF membrane. After blocking for 1 h with 5% skim milk in TBST, membranes were incubated overnight at 4 °C with antibodies against β-actin (MAB1501 Millipore), LC3B (L7543 Sigma), Beclin-1 (G-11 Santa Cruz Biotechnology), p62 (D-3 Santa Cruz Biotechnology), AMPK (#5831 Cell Signalling), phospho-AMPK (#2531 Cell Signaling), CHOP/GADD153 (H5- Santa Cruz Biotechnology), eIF2a (#9722 Cell Signaling), phospho-eIF2a (Ser51) (#9721 Cell Signalling) and cleaved caspase-8 (Asp387) (#8592 Cell Signalling). Membranes were then probed with goat anti-mouse IgG-HRP (31430, Thermo Scientific) or with goat anti-rabbit IgG-HRP conjugate (12–348, Millipore) secondary antibodies at room temperature for 1 h. An aliquot of pooled standard sample was loaded in one lane of each gel. The pooled sample served as an internal standard to minimize the inter-assay variation for samples run in different gels. Detection of the immuno-reactive bands was performed using the Clarity Western ECL Substrate (BioRad). β-actin served as a loading control. Densitometric analysis was performed using Image J software (NIH, Bethesda, MD, USA).

### 4.7. Statistical Analysis

Data are presented as mean values ± standard deviation (SD) and percentages, unless stated otherwise. Student’s *t*-test, Welch’s test or Mann–Whitney test were used, as appropriate, for comparisons of quantitative variables (body weight and biochemical parameters) between the Empa- and control animal groups. Normality of distribution for these variables was tested with the Shapiro–Wilk test and the equality of variances with the Levene’s test. Comparisons between groups for qualitative variables were performed with the Fisher’s exact test, as appropriate. Within each group, paired t-tests were used to compare levels of each studied parameter before and after the five-week intervention period. A *p* value < 0.05 was considered statistically significant. All statistical analyses were performed using GraphPad Prism Software (v.7) (Graph Pad software, San Diego, CA, USA).

## 5. Conclusions

We provide novel evidence that empagliflozin treatment for five weeks in HFD-fed ApoE^(-/-)^ mice can attenuate NAFLD by not only improving metabolism and inflammation progression but also by promoting autophagy, reducing HFD-induced ER stress and inhibiting hepatocyte apoptotic processes. Further research studies with longer duration and various empagliflozin doses are required to expand on our present findings and further delineate possible dose and duration-dependent differential effects of empagliflozin on NAFLD development and progression. Clarifying the precise molecular mechanisms underpinning the empagliflozin-induced increase in autophagic flux in hepatocytes will advance our understanding regarding the role of empagliflozin as a potential therapeutic option for NAFLD/NASH prevention and/or treatment.

## Data Availability

Data is contained within the article and its supplementary material.

## Supplementary Materials

The following are available online at https://www.mdpi.com/1422-0067/22/2/818/s1, Data S1: The effect of empagliflozin on atherosclerotic plaque formation; Figure S1: Atherosclerotic plaque and mRNA expression of molecules implicated in atherogenesis in ApoE^(-/-)^ treated with either 10 mg/kg/day Empagliflozin (Empa-group) or vehicle (control-group) for 5 weeks; Table S1: List of primer sequences used for RT-PCR analysis in this study.

## Abbreviations

ApoE	Apolipoprotein E
ATF4	Activating Transcription Factor 4
AΤF6	Activating Transcription Factor 6
ALT	Alanine aminotransferase
AST	Aspartate aminotransferase
ACLY	ATP citrate lyase
Acc2	Acetyl coA carboxylase 2
AMPKa1	AMP-activated protein kinase alpha 1
AMPKa2	AMP-activated protein kinase alpha 2
Bcl-2	B-cell lymphoma 2
Bax	BCL2-associated X protein
CHOP	C/EBP homologous protein
CVD	Cardiovascular disease
Dgat2	Diacyl glycerol acetyltransferase 2
DMT2	Diabetes mellitus type 2
Empa	Empagliflozin
ER	Endoplasmic reticulum
elF2α	Eukaryotic Initiation Factor 2 alpha
Fasn	Fatty acid synthase
F4/80	EGF-like module-containing mucin-like hormone receptor-like 1
FFA	Free fatty acid
GRP78	Binding immunoglobulin protein
GRP94	Glucose-regulated protein 94
GPAT	Glycerol-3-phosphate acyltransferase
HFD	High-fat diet
HbA1c	Hemoglobin A1c
ICAM-1	Intercellular adhesion molecule-1
Il-6	Interleukin 6
Insig	Insulin-induced gene
IRE1α	Inositol-requiring enzyme 1 α
LC3B	Microtubule-Associated Protein 1 Light Chain 3B
mTOR	Mechanistic Target Of Rapamycin Kinase
Mcp-1	Monocyte chemoattractant protein-1
NAFLD	Non-alcoholic fatty liver disease
NASH	Non-alcoholic steatohepatitis
NAS	NAFLD Activity Score
Ppar-γ	Peroxisome proliferator-activated receptor gamma
Pck-1	Phosphoenolpyruvate carboxykinase 1
p62(Sqstm1)	Sequestosome 1
qPCR	Quantitative polymerase chain reaction
ROS	Reactive oxygen species
SGLT-2i	Sodium-glucose cotransporter-2 inhibitors
Screbp-1	Sterol regulatory element-binding protein 1
Scd-1	Stearoyl-CoA desaturase 1
SGOT	Glutamic oxaloacetic transaminase
SGPT	Glutamic-pyruvic transaminase
TNF-α	Tumor necrosis factor alpha
UPR	Unfolded protein response
VCAM-1	Vascular cell adhesion molecule-1
ΧΒΡ1	X-box binding protein 1
ZDF	Zucker diabetic fatty
γ-GT	Gamma-glutamyl transferase
