# Supplementary material for: Empagliflozin Attenuates Non-Alcoholic Fatty Liver Disease (NAFLD) in High Fat Diet Fed ApoE(-/-) Mice by Activating Autophagy and Reducing ER Stress and Apoptosis
Source: Int J Mol Sci. 2021 Jan 15;22(2):818. doi: 10.3390/ijms22020818 (PMC7829901; doi:10.3390/ijms22020818)
Supplement: Supplementary file 1 [file ijms-22-00818-s001.pdf]

Supplementary Materials

Data S1. The effect of empagliflozin on atherosclerotic plaque formation.

Background

It has been shown that patients with severe NAFLD have a 2- to 3-fold increased risk of developing T2D and CVD events [1,9] while empagliflozin, an antidiabetic agent, treatment for 3.1 years (median time) in type 2 diabetes patients with prevalent CVD, lowered the combined CVD endpoint of CVD death, non-fatal stroke and non-fatal myocardial infarction [11]. Interestingly, the observed cardio-protective effect of empagliflozin was irrespective of glycemic control [8]. To this end, we also investigated the effect of this treatment on atherogenesis, focusing on the expression of pro-inflammatory and adhesion molecules that promote the atherogenic process.

Methods

Quantification of atherosclerotic lesion area

At the end of the 5-weeks of treatment, all mice were sacrificed under isoflurane anesthesia by transection of the diaphragm and the aorta was rapidly removed, fixed and embedded in paraffin. 4-μm-thick sections were stained with hematoxylin–eosin (H&E) and used for histopathological analysis. The degree of pathological changes was evaluated microscopically by measuring the area of atherosclerotic plaques. Results are reported as the percentage of the neointima area containing the lesion. Plaque area analysis was carried out using Image Pro Plus software version 5.1 (Media Cybernetics, Inc.).

qPCR analysis:

The mRNA levels of adhesion molecules *Icam-1* and *Vcam-1*, and inflammatory markers *Il-6, Tnf-α, F4/80*, *Mmp-2* and *Timp-1* were also evaluated in aortic tissues using qPCR assay.

Results

Empagliflozin administration for 5 weeks did not significantly affect the atherosclerotic plaque formation

Two out of 8 mice in the Empa-group developed aortic atherosclerotic plaque lesions, while in the control-group three out of 8 mice presented with such atherosclerotic plaques (representative Figure S1.a). The mean atherosclerotic lesion area (as measured by the percentage of lumen area covered by total plaque area in all aortic root sections) was lower in the Empa-group, albeit not significantly ($p = 0.17$) (Figure S1.b).

Empagliflozin administration for 5 weeks reduced the expression of adhesion and inflammatory molecules and improved matrix metalloproteinase profile

The effect of the 5-week empagliflozin treatment on the expression of inflammatory molecules (*IL-6, TNF-α, F4/80*), adhesion molecules (*ICAM-1, VCAM-1*), matrix metalloproteinase (*MMP-2*) and its inhibitor (*TIMP-1*) was evaluated by qRT-PCR. Our results showed that, the 5-week empagliflozin administration significantly reduced IL-6 and MMP-2 mRNA levels ($p = 0.045$ and $p = 0.048$, respectively) (Figures s1.c and s1.e), whilst marginally decreased the *TNF-α* and *VCAM-1* mRNA levels ($p = 0.077$ and $p = 0.072$, respectively) (Figures S1.c&d).

Empagliflozin treatment had no significant effect on *F4/80, Icam-1* and *Timp-1* mRNA levels (Figures S1.c,d,e). The mRNA *Timp-1/Mmp-2* ratio (the balance between *MMPs* and *TIMPs* is a known indicator of *MMPs* overall collagenolytic activity) at the end of the 5-week empagliflozin treatment was found to be higher in Empa-group compared to control-group, approaching significance ($p = 0.05$) (Figure S1.f).

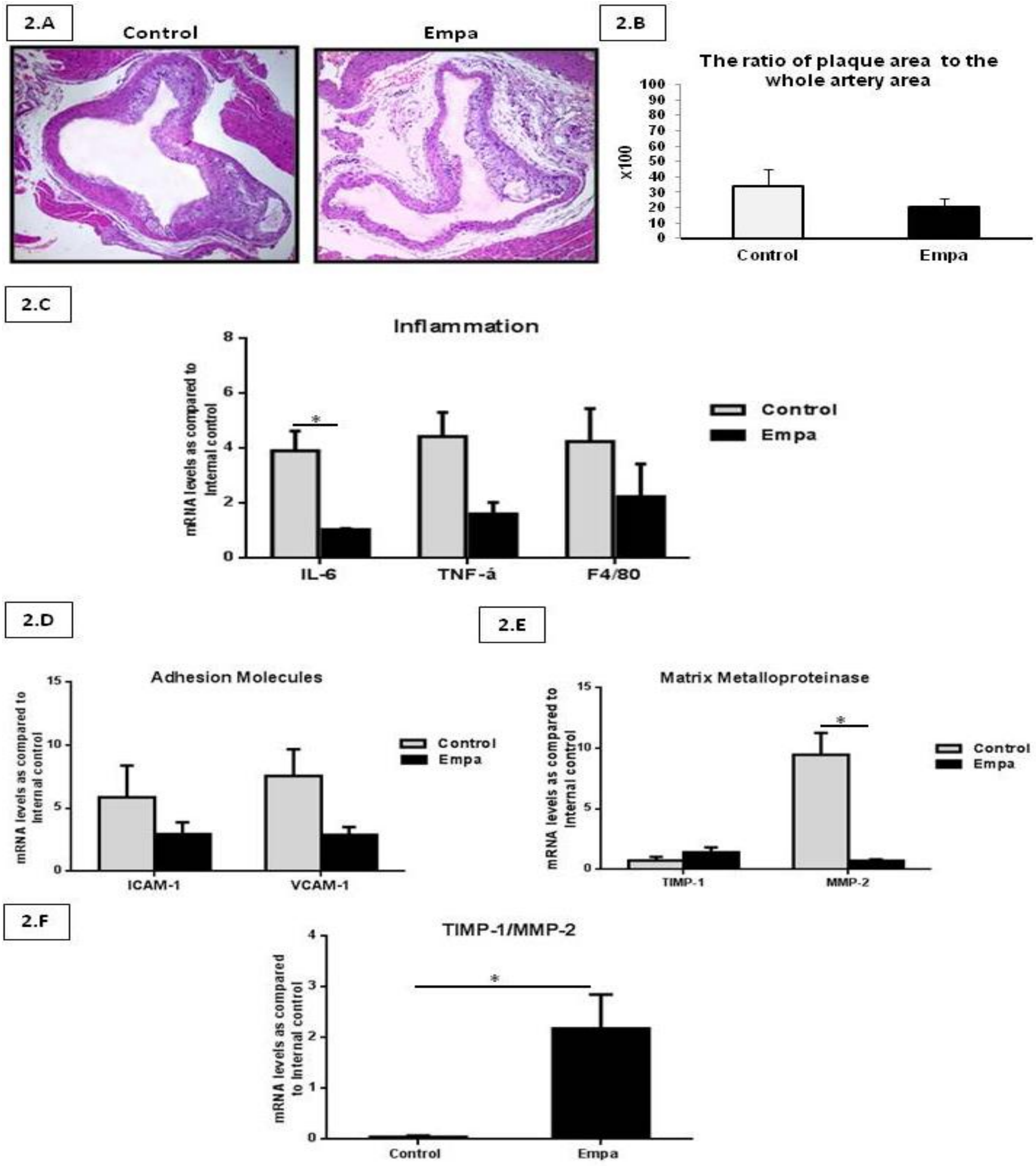

Figure S1. Atherosclerotic plaque and mRNA expression of molecules implicated in atherogenesis in ApoE$^{(-/-)}$ treated with either 10 mg/kg/day Empagliflozin (Empa-group) or vehicle (control-group) for 5 weeks. **a.** Selected 4 μm section images from the aortic root stained with H&E (Original magnification ×40).**b.** Quantification of plaque area is shown as a percentage of lumina stenosis by thickened intima. Values are shown as mean ± SD. **c.** *IL-6, TNF-α* and *F4/80* (inflammatory markers) mRNA expression in thoracic aorta tissues of mice. *IL-6* mRNA expression was significantly decreased in Empa-group as compared to control-group. **d.** *ICAM -1* and *VCAM-1* (adhesion molecules) mRNA expression in thoracic aorta tissues of mice. A reduced expression of *VCAM-1* in Empa-group approaching significance ($p = 0.072$), as compared to control group. **e.** The mRNA levels of *MMP-2* and its inhibitor *TIMP-1* (gelatinolytic activity) in thoracic aorta tissues. *MMP-2* mRNA expression was significantly decreased in Empa-group compared to control-group. **f.** *TIMP-1/MMP-2* mRNA ratio was also significantly increased in Empa-group as compared to control-group. qPCR data are shown as mean ± SEM. * : $p < 0.05$.

Table S1. List of primer sequences used for RT-PCR analysis in this study.

Primer	Forward	Reverse
18S	5’-GTTCCGACCATAAACGATGCC-3’	5’-TGGTGGTGCCTTCCGTCAAT-3’
mTOR	5’- CCATCCAATCTGATGCTGGA-3’	5’- GGTGTGGCATGTGGTTCTGT-3’
LC3β	5’-GACGGCTTCCTGTACATGGTTT-3’	5’-TGGAGTCTTACACAGCCATTGC-3’
p62	5’- TGTGGAACATGGAGGGAAGAG-3’	5’- TGTGCCTGTGCTGGAACTTTC-3’
Beclin-1	5’- GTGCGCTACGCCCAGATC-3’	5’- GATGTGGAAGGTGGCATTGAA-3’
AMPKa1	5’- GTCAAAGCCGACCCAATGATA-3’	5’- CGTACACGCAAATAATAGGGGTT-3’
AMPKa2	5’- CAGGCCATAAAGTGGCAGTTA-3’	5’- AAAAGTCTGTCGGAGTGCTGA-3′
Bcl-2	5’-TTCAGGGATGGGGTGAACTG-3’	5’-CACAGGGCGATGTTGT-3’
Bax	5’- CCCGAGAGGTCTTCTTCC-3’	5’- GCCTTGAGCACCAGTTTG-3’
CHOP	5´-CCACCACACCTGAAAGCAGAA-3´	5´-GGTGCCCCCAATTTCATCT-3´
GRP78	5´-ACATGGACCTGTTCCGCTCTA-3´	5´-TGGCTCCTTGCCATTGAAGA-3´
Xbp1	5´-ACATCTTCCCATGGACTCTG-3´	5´-TAGGTCCTTCTGGGTAGACC-3´
GRP94	5´-TTGTGTCCAATTCAAGGTAATCA-3´	5´-TTGCTGACCCAAGAGGAAAC-3´
eIF2a	5´-CAACGTGGCAGCCTTACA-3´	5´-TTTCATGTCATAAAGTTGTAGGTTAGG-3´
ATF4	5´-AGCCCCACAACATGAC-3´	5´-CCACCTCCAGATAGTCAT-3´
ATF-6	5´-GATGCAGCACATGAGGCTTA-3´	5´-CAGGAACGTGCTGAGTTGAA-3´
IRE1	5´-CTGTGGTCAAGATGGACTGG-3´	5´-GAAGCGGGAAGTGAAGTAGC-3´
Fasn	5´-GGAGGTGGTGATAGCCGGTAT-3´	5´-TGGGTAATCCATAGAGCCCAG-3´
Scd-1	5´-ACGCCGACCCTCACAATTC-3´	5´-CAGTTTTCCGCCCTTCTCTTT-3´
Acaca	5´-GTCCCCAGGGATGAACCAATA-3´	5´-GCCATGCTCAACCAAAGTAGC-3´
Screbp-1	5´-CGGAAGCTGTCGGGGTAG-3´	5´-GTTGTTGATGAGCTGGAGCA-3´
Pck-1	5´-CCACAGCTGCTGCAGAACA-3´	5´-GAAGGGTCGCATGGCAAA-3´
Ppar-γ	5′- TAGGTGTGATCTTAACTGCCG-3′	5′- GCATCGTGTAGATGATCTCA -3′
TNF-α	5´-ACCCTCACACTCAGATCATCTTC-3´	5´-TGGTGGTTTGCTACGACGT-3´
IL-6	5´-GAGGATACCACTCCCAACAGACC-3´	5´- AAGTGCATCATCGTTGTTCATACA-3´
F4/80	5´-CTTTGGCTATGGGCTTCCAGTC-3´	5´-GCAAGGAGGACAGAGTTTATCGTG-3´
SGLT-1	5´-AAGATCCGGAAGAAGGCAT-3´	5´-GCGGAGGTACTGAGGCATTGTG-3´
SGLT-2	5´-GTTCCGACCATAAACGATGCC-3´	5´-TGGTGGTTGCCCTTCCGTCAAT-3´
ICAM-1	5´-GGTTCTCTGCTCCTCCACAT-3´	5´-CCTTCCAGGCTTTCTCTTTG-3´
VCAM-1	5´-CTTCCAGAACCCTTCTCAG-3´	5´-GGGACCATTCCAGTCACACTTC-3´
TIMP-1	5'-GCATGGACATTTATTCTCCACTGT-3'	5'-TCTCTAGGAGCCCCGATCTG-3'
MMP-2	5'-CCCTCAAGAAGATGCAGAAGTTC-3'	5'-TCTTGGCTTCCGCATGGT-3'
